# The Effectiveness of Health Education Using Audiovisual on the *Santri* Smokers’ Motivation to Stop Smoking

**DOI:** 10.31557/APJCP.2021.22.8.2357

**Published:** 2021-08

**Authors:** Ismail Ismail, Rapitos Siddiq, Bustami Bustami

**Affiliations:** 1 *Department of Nursing, Polytechnic of Health-Ministry of Aceh, Aceh, Indonesia. *; 2 *Department of Nursing, Polytechnic of Health-Ministry of Padang, Padang, Indonesia. *

**Keywords:** Health education, audiovisual, cancer sufferers due to smoking, motivation to quit smoking

## Abstract

**Background and objective:**

Low motivation to quit smoking affects individual smoking behavior. Health education using audiovisual media can increase smokers’ motivation to quit smoking. The objective of this study was to determine the effectiveness of health education using audiovisuals on the Santri smokers’ motivation to stop smoking.

**Methods:**

This quasi-experimental study was carried out using a pretest-posttest design. The sample in this study included Santri smokers studying at the Traditional Islamic Boarding School in Aceh Besar . This study consisted of 4 groups. Three groups were given intervention (audiovisual health education with different themes) and one group received just health education. Groups were compered in terms of difference in the mean smokers’ motivation to quit smoking. The data analysis was done by running paired t-test and one-way ANOVA.

**Results:**

The results of statistical tests showed that there was a difference in the mean motivation to quit smoking before and after the intervention in each group (mean ± SD for group 1 to 4 was 11.52 ± 4.76, 15.39 ± 6.06, 22.57 ± 6.23, and 9.84 ± 6.42, respectively). The highest increase in the mean motivation to quit smoking was allocated to group 3 who received audiovisual health education with the theme of risk of developing cancer due to smoking.

**Conclusion:**

Health education using audiovisuals could increase the motivation of students to quit smoking, especially interventions on the risk of developing cancer due to smoking. Therefore, health workers are suggested to use audiovisuals to implement various intervention in order to change smoking behavior in students.

## Introduction

The smoking behavior of adolescents is very concerning. Smoking behavior greatly impacts the lungs, increases the risk of several diseases, and reduces the health of smokers in general (Fajar, 2011; Courtney, 2015). Smoking behavior has become a habit that is often misunderstood in everyday life. Various factors can cause a person to start smoking, one of which is environmental factors (Prabandari, 2018).

According to WHO (2020), Indonesia has the world’s highest prevalence of smoking (76.2%), followed by Jordan (70.2%), Kiribati (63.9%), and Sierra Keone (60%). Based on Indonesia Basic Health Research (RISKEDAS), the prevalence of smoking in the population aged 10-18 years was 7.2 % in 2013, and increased to 9.1% in 2018 (Kemenkes, 2018).

Every effort has been made to reduce the prevalence of smoking, such as increasing cigarette taxes, advertising and forming educational campaigns, banning smoking in public places, holding smoking cessation therapy, and displaying pictorial health warnings (PHW) on cigarette packs (Gilbert and Cornuz, 2003; Golechha, 2016). To reduce smoking behavior in adolescents, the government also establishes smoking prohibition regulations and smoke-free areas in school environments (Sulistiadi et al., 2020). However, the prevalence of smoking among adolescents continues to increase, especially among adolescents in traditional Islamic boarding schools that do not have smoking prohibition regulations.

Health education is an effort that can be made to change smoking behavior. The negative beliefs about smoking among low-educated adolescents increased after health education. The results of a research conducted by Graff et al., (2016) showed that the video version was more effective for educating low-educated adolescents about smoking .

The anti-tobacco message using audiovisuals significantly increased participants’ knowledge on smoking hazards and changed their attitudes towards tobacco use in north India (Kaur et al., 2012). Other studies revealed that video-based computer-tailored smoking cessation interventions were more effective for smokers to achieve long-term abstinence than brief generic text advice (Stanczyk et al., 2016). Health education using audiovisuals more effectively changed adolescents’ attitudes toward the dangers of smoking (Siregar and Sandika, 2019). 

During language learning process, the use of audiovisuals makes the class interactive and interesting, motivates students, and facilitates learning of language skills (Al Mamun, 2014). It is also hoped that using audiovisuals might contribute to smokers’ efforts to change bad health habits and having healthier lifestyles. Providing material with different themes and using audiovisual media had different results on smoking behavior. For this reason, we aimed at discovering the effectiveness of health education using audiovisuals on the Santri smokers’ motivation to stop smoking (Santri are students who study in Islamic boarding schools .

## Materials and Methods

This quasi-experimental study used a pretest-posttest design to investigate the effectiveness of health education using audiovisuals on the Santri smokers’ motivation to stop smoking. For this purpose, Santri smokers aged 15-19 years old were selected from 4 traditional Islamic boarding schools in Aceh Besar. For sampling, two-stage sampling design was used. At the first stage, clusters random sampling and at the second stage purposive sampling were applied. This study consisted of 4 groups. Three groups were given intervention (audiovisual health education with different themes) and one group received just health education. Each group included 38 participants. This research was conducted from February 3 to February 24, 2020. Informed consent was obtained from all the participants involved in the study.

Data collection was carried out by distributing Motivation to Quit Smoking Questionnaire adopted from a research by Putri (2015). The questionnaire consists of 39 statements and is scored using a 4-point Likert scale (strongly agree, agree, disagree, and strongly disagree). 

The point given to each response depends on whether the statement is positive or negative. In case of positive, the order of scoring is reversed from 4 as strongly agree to 1 as strongly disagree. In case of negative, the order of scoring is reversed from 1 as strongly agree to 4 as strongly disagree. This questionnaire has been tested for validity and reliability having a Cronbach’s alpha value of 0.938.

The questionnaire was completed by the participants s 1 day before receiving the intervention. The intervention was carried out 2 times for each group at 1-week interval. The questionnaire was also re-completed by the participants 1 day after receiving the second intervention.. An average of 20 minutes was considered for filling the questionnaire.

**Figure 1:**
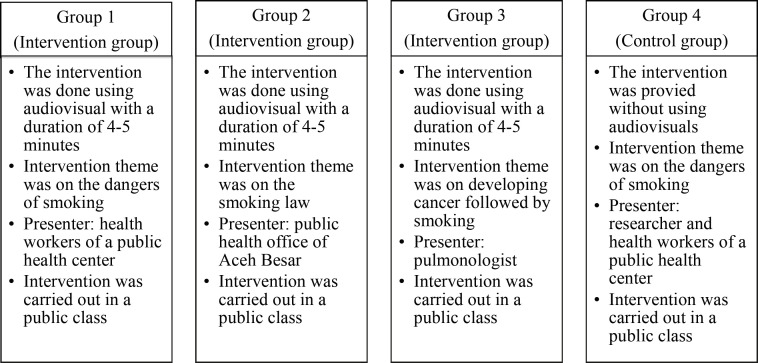
The Intervention Theme for Each Group

The intervention theme was on the risks of smoking for group 1, smoking law for group 2, and risk of developing cancer caused by smoking for group 3. Participants received health education intervention using audiovisuals in a room and given and each video lasted between 4-5 minutes. Further explanation was provided to the participants by 3 experts, namely health workers of the public health center, the health promotion staff of the Aceh Besar health office, and pulmonologists. The control group only received health education on the risks of smoking, which was delivered by the researchers and assisted by health workers at the health center. This session lasted between 15-20 minutes (Figure 1)

For data analysis, univariate and bivariate analysis were performed using SPSS . The data of the study were normally distributed, so mean and standard deviation were used to present the data. For the comparison of groups, , paired t-test and one-way ANOVA were run. 

## Results

*Characteristics of the participants*

Table 1 shows that the median age of Santri smokers in groups 1, 2, and 3 was 17, 17, and 16 years old, respectively. Based on the statistical test, there was no difference among three intervention groups with respect to age (p-value = 0.081). The median length of being a Santri in group 1, 2, 3, and 4 was 3 years old. Based on the statistical test, there was no difference among three intervention groups with respect to length of time they were a Santri (p-value = 0.097).

*The mean motivation to quit smoking before and after intervention*

As depicted from Table 2, there was an increase in the mean motivation to quit smoking in group 1 with a mean score of 11.52 and a standard deviation of 4.76. The results of statistical tests showed that there was a significant difference in the mean motivation to quit smoking before and after the intervention in group 1.

In group 2, there was an increase in the mean motivation to quit smoking with a mean score of 15.39 and a standard deviation of 6.06. The results of statistical tests showed that there was a significant difference between the mean motivation to quit smoking before the intervention and after the intervention in group 2.

In group 3, there was an increase in the mean motivation to quit smoking with a mean score of 22.57 and a standard deviation of 6.23. The results of statistical tests showed that there was a significant difference between the mean motivation to quit smoking before the intervention and after the intervention in group 3.

In the control group, there was an increase in the mean motivation to quit smoking with a mean score of 9.84 and a standard deviation of 6.42. The results of statistical tests showed that there was a significant difference between the mean motivation to smoke before the intervention and after the intervention in the control group.

Before the intervention, there was no difference in the mean motivation to quit smoking in none of the groups with a p-value of 0.920. After the intervention, there was a difference in the mean motivation to quit smoking in all of the groups with a p-value of 0.000. The increase in the mean motivation to quit smoking was highest in group 3 who were given health education using audiovisuals with the theme of risk of developing cancer caused by smoking.

To determine which intervention group had different meanings between the three groups , post hoc tests using Tukey HSD were carried out. Based on Table 3. the mean change in the motivation score to quit smoking in group 1 had a different meaning from group 2 and group 3 or vice versa. In addition, changes in the mean score of motivation to quit smoking in group 1 differed in meaning from the control group or vice versa.

**Table 1:** Characteristics of Santri Respondents in Each Group

Characteristics	Group 1	Group 2	Group 3	Group Control	p-value †
	(Median; Range)	(Median; Range)	(Median; Range)	(Median; Range)	
Age (year)	17; 15-19	17; 15-19	16; 15-19	17; 15-19	0.081
Length Being of Santri (year)	3; 2-5	3; 2-5	3; 2-5	3; 2-5	0.097

†, Kruskal wallis Test. P.value<0.05

**Table 2:** Differences in Average Motivation to Quit Smoking Before and After Intervention

	Groups	Group 1	Group 2	Group 3	Group Control	p-value‡
		(Mean ± SD)	(Mean ± SD)	(Mean ± SD)	(Mean ± SD)	
Motivation	Pre-Test	88.21 ± 2.24	88.23 ± 2.40	88.60 ± 3.50	88.36 ± 2.61	0.920
	Post-Test	99.73 ± 4.67	103.63 ± 5.87	111.18 ± 5.52	98.21 ± 5.90	0.000
	p-value†	0.000	0.000	0.000	0.000	
	Changes	11.52 ± 4.76	15.39 ± 6.06	22.57 ± 6.23	9.84 ± 6.42	0.000

†, paired t-test. P.value<0.05; ‡, one way ANOVA. P.value<0.05

**Table 3:** Post Hoc Test Results on Difference in Smoking Motivation Between Each Intervention Group

(I) Kelompok		(J) Kelompok	∆Mean (I-J)	p - value	CI 95%
Tukey HSD	Group 1	Group 2	-3.86	0.025	-7.38 – -0.34
		Group 3	-11.05	0.000	-14.57 – -7.53
		Control Group	1.68	0.601	-1.83 – 5.31
	Group 2	Group 1	3.86	0.025	0.34 – 7.38
		Group 3	-7.18	0.000	-10.71 – -3.66
		Control Group	5.55	0.000	2.03 – 9.07
	Group 3	Group 1	11.05	0.000	7.53 – 14.57
		Group 2	7.18	0.000	3.66 – 10.71
		Control Group	12.73	0.000	9.21 – 16.25
	Control Group	Group 1	-1.68	0.601	-5.21 – 1.83
		Group 2	-12.73	0.000	-9.07 – -2.03
		Group 3	-3.86	0.000	-16.25 – -9.21

## Discussion

Recognizing smoking motivation is a key point to help prevent students from starting smoking and help them to quit smoking (Kim, 2018). Smoking addiction is positively related to the level of motivation to quit smoking. Addiction is also positively associated with smoking cessation efforts in the past year but is not related to future attempts to quit smoking and achieve successful abstinence (Perski et al., 2019). 

Previous research results indicated that increase of motivation would help smokers to quit smoking (Ussher et al., 2016; Kleier, 2017). Higher motivation to quit smoking results in a more decrease in smoking behavior (Julina, 2017). Increasing one’s motivation can be done by providing health education. Health promotion is pivotal in the drive to reduce the growing burden of chronic disease worldwide due to tobacco and particularly smoking. Comprehensive and active awareness of the population through the health promotion strategies are the primary tools for smoking prevention and cessation. Public education is an integral part of the efforts to both prevent the initiation of smoking use and encourage smoking cessation. Increased health promotion efforts about the detrimental health effects from smoking use may result in higher levels of knowledge about the harms of smoking and this in turn could increase quit intentions and subsequent quitting among users . By increasing their knowledge about smoking cessation methods, health professionals can support and encourage the large majority of smokers who want to quit (Golechha, 2016).

Health education using audiovisuals was more effective in increasing the motivation to quit smoking compared to health education alone according to our findings. Audiovisuals improve one’s critical and analytical thinking. They are memorable, thought-provoking, stimulating, and appealing to many people. Through audiovisuals, a person can present videos, programs, and films in a more effective way since it helps retain information and makes the concepts better about smoking and the dangers of smoking (Emen and Edrada, 2020).

Our results showed that there was a difference in the mean motivation to quit smoking after the intervention in all groups. The increase in the mean motivation to quit smoking was highest in group 3 who were given health education using audiovisuals with the theme of risk of developing cancer caused by smoking.

The warning to stop smoking in group 3 who was given a video about the story of a patient who had laryngeal cancer due to smoking increased the student’s motivation to quit smoking more than the first intervention . Similarly, Borland and Hill (1997) showed that awareness of cigarette knowledge and belief in smoking about health effects increased after a new warning.

The experience of family members suffering from cancer increases family awareness about cancer and changes life habits to become healthier. This happened after being given the second intervention, where the motivation of the students to quit smoking increased after being given a video of the experience of someone suffering from laryngeal cancer due to smoking (Hikmatia and Zahra, 2018) . 

The most common smoking cessation message is showing pictures of damaged lungs or people with nasopharyngeal cancer. This pictorial message causes smokers to dislike smoking cessation ads because it too clearly shows the diseases caused by smoking (Sibarani and Perbawaningsih, 2018). Pictorial health warnings on cigarette packaging can increase smokers’ motivation to quit smoking (Novarianto, 2015; Layoun et al., 2017).

The experience of cancer patients who developed cancer following smoking is a clear evidence of smoking consequences. When the video was screened, the researchers observed that some students felt uneasy about smoking cigarettes. Several questions arose by the smokers on the contents of cigarettes which can cause cancer. Santri smokers also asked following questions: ”How long does it take to get cancer following smoking?” and “How to quit smoking?”. 

The results of the present study also showed receiving health education on the smoking law increased the participants motivation to quit smoking in group 2. In the teachings of Islam, if a person smokes and causes certain harm to himself (muhaqqah), then that person is prohibited from smoking. 

A tobacco fatwa is a fatwa (Islamic legal pronouncement) that prohibits tobacco usage by Muslims. Scholars have also a complementary role in reducing tobacco use and even motivating smokers to quit smoking (Aliya et al., 2017). A research was conducted by Alzyoud et al., (2015) on 950 Muslim students. They showed 32% of the [participants were regular daily tobacco users and 72% prayed regularly. Prayer frequency was negatively associated with tobacco smoking. Religious observance was found to be strongly associated with tobacco smoking among Jordanian youth. 

Statistically, the attitude of adolescents towards the fatwa on smoking does not correlate with smoking cessation behavior. However, the more positive the respondent’s attitude towards smoking haram fatwa, the more positive the smoking behavior. The more negative the students’ attitudes towards the fatwa on smoking haram, the student’s smoking behavior becomes positive, so that many students have quit or reduced smoking intensity, but there are still some Muslim students who still smoke (Zakiyah et al., 2016).

The results of the post hoc test showed that there was no difference in the mean motivation to quit smoking between group 1 and the control group, suggesting that mean motivation to quit smoking after health education intervention using audiovisuals with the theme of the dangers of smoking was not different from mean motivation to quit smoking after receiving health education only.

In a nutshell, according to the findings of this study, provision of health education using audiovisuals was more effective for increasing the smokers ‘motivation to quit smoking compared to provision of health education alone. 
